# CREM Is Correlated With Immune-Suppressive Microenvironment and Predicts Poor Prognosis in Gastric Adenocarcinoma

**DOI:** 10.3389/fcell.2021.697748

**Published:** 2021-12-06

**Authors:** Kuai Yu, Linju Kuang, Tianmei Fu, Congkai Zhang, Yuru Zhou, Chao Zhu, Qian Zhang, Zhanglin Zhang, Aiping Le

**Affiliations:** ^1^ Department of Blood Transfusion, The First Affiliated Hospital of Nanchang University, Nanchang, China; ^2^ Key Laboratory of Jiangxi Province for Transfusion Medicine, The First Affiliated Hospital of Nanchang University, Nanchang, China

**Keywords:** CREM, exhausted T cell, tumor-associated macrophages, tumor-infiltrating, immunosuppression, gastric cancer tumor microenvironment, prognosis

## Abstract

The transcriptional repressor cAMP response element modulator (CREM) has an important role in T-cell development. In this study, we used the integrated Bioinformatics Methods to explore the role of CREM in gastric adenocarcinoma (GAC). Our results showed that high CREM expression was closely related with poorer overall survival in GAC. By GSEA cluster analysis, we found that the high expression of CREM was associated with the cancer-associated pathway in GAC. Moreover, single-cell sequencing data showed that CREM is mainly localized in exhausted CD8^+^ T cells. Its prognostic value and the potential function lead to T-cell exhaustion in the tumor microenvironment (TME). Similar results were also obtained in glioma and lung cancer. High expression of CREM, correlated with clinical relevance of GAC, was associated with T-cell exhaustion and M2 polarization in GAC. These findings suggest that CREM can be used as a prognostic biomarker in GAC, which might provide a novel direction to explore the pathogenesis of GAC.

## Introduction

Gastric adenocarcinoma (GAC), which arises from glandular epithelia of the gastric mucosa, is the most common type of gastric cancer, which is the fourth most common cancer, and the second most common cause of cancer death in the world (Bray et al., 2018). At present, the first-line treatment of advanced GAC is still chemotherapy (Song et al., 2017). Monoclonal antibodies targeting human epidermal growth factor (HER2; trastuzumab) (Zaanan et al., 2018) have been approved by the FDA for first-line treatment of patients with HER2-positive GAC. In addition, Ramucirumab, a vascular endothelial growth factor (VEGF) targeted drug (Yazici et al., 2016), for whom first-line treatment has failed, has also been approved for patients with advanced GAC. Although there are many treatments for GAC, the overall survival rate is only 5–20% in the world.

Immunotherapy is a revolutionary anti-cancer therapy in the past decade. Immune checkpoint inhibitors, which are closely related to the elimination of T-cell exhaustion, have achieved certain results in the treatment of various tumors. The most widely used checkpoint inhibitors are antibodies of cytotoxic T lymphocyte antigen 4 (CTLA-4), programmed cell death protein 1 (PD-1), and its ligand programmed death-ligand 1 (PD-L1) (Fuchs et al., 2014). However, current immunotherapies, such as anti-CTLA4 (Fuchs et al., 2014; Li et al., 2019), showed poor clinical efficacy in GAC; in addition, anti-PD-1 and anti-PD-L1 showed a partial response in GC. With the dramatic development of next-generation sequencing (NGS) and single-cell sequencing, more and more exhaustion T-cell-related molecules have been identified, such as TIM3, LAG3, TIGIT (Anderson et al., 2016), PD1 (Xing et al., 2018), and LAYN (Zheng et al., 2017). The identification of these molecules is of great significance in predicting the prognosis of cancer prognosis. Because the existing marker is still unable to effectively identify exhaustion T cells, this field requires new biomarkers as prognostic indicators to effectively enhance prognosis and individualized immunotherapy.

The transcriptional repressor cAMP response element modulator (CREM) is inducible by activation of the cAMP signaling pathway with the kinetics of an early response gene (Lamas et al., 1996). The CREM gene encodes both activators and repressors of cAMP-dependent transcription. Previous studies have shown that cyclic AMP-dependent signals are associated with T-cell exhaustion (Maine et al., 2016; Schmetterer et al., 2019). Moreover, CREM contributes to various cellular and molecular abnormalities in T cells, including increased IL-17 and decreased IL-2 expression (Verjans et al., 2013). Previous studies have shown that CREM has important roles in normal T-cell physiology and contributes to aberrant T-cell function in patients with systemic lupus erythematosus (SLE) (Rauen et al., 2011). RNAi targeting of ICER/CREM in responder CD25^–^ CD4^+^ T cells antagonizes Treg-mediated suppression (Bodor et al., 2007). These findings suggest that CREM has multifaceted functional roles in immune microenvironment. However, the underlying functions of CREM in TME is still unclear.

This study aims to delineate the role of CREM, through TCGA GAC public data and TISCH single-cell database. We analyzed the expression of CREM and the correlation with prognosis of GAC patients in the TCGA database. Through KOBAS database and GSEA analysis, we found that the high expression of CREM is closely related to cancer-associated pathways. To investigate the underlying causes, we investigated the correlation of CREM with tumor-infiltrating immune cells in the tumor microenvironment (TME) *via* CIBERSORT. Our report depicted the important role of CREM in GAC, and revealed the relationship between CREM and tumor-infiltrating lymphocytes in GAC. These results suggest that CREM may be a potential clinical prognostic indicator in GAC. In addition, we briefly explored the role of CREM in lung cancer and glioma.

## Methods

### Data Download and Processing

The mRNA profiling information is from the TCGA (The Cancer Genome Atlas, https://cancergenome.nih.gov/) database. The download mRNA expression data were alternate with log2, so we restored them to raw FPKM (Fragments Per kilobase per Million) and count value. Besides, we changed FPKM gene expression value to TPM (Transcripts Per Million) expression value. There were 375 GAC patients in mRNA expression data and were listed in descending order by CREM TPM expression value. Finally, we selected the top 98 patients as CREM high expression group (CREM TPM expression value >9) and the last 44 patients as CREM low expression group (CREM TPM expression value <4).

### CREM Gene Expression Analysis in Bulk and Single-Cell RNA Sequence Datasets

The CREM expression level was analyzed by the GEPIA2 and TISCH web-based tools. GEPIA2 contained TCGA and GTEx datasets (Tang et al., 2019). TISCH (Tumor Immune Single-cell Hub) provided detailed cell-type annotation and gene expression profile at the single-cell level (Sun et al., 2021). We invested CREM single-cell gene expression level in the GSE134520 (Zhang et al., 2019) GAC dataset and the GSE131928 (Neftel et al., 2019) glioma dataset. T-cell single-cell dataset of non-small cell lung cancer was analyzed in web database (http://lung.cancer-pku.cn) (Guo et al., 2018).

### Differentially Expressed Genes About CREM

In this study, we explored differential expression genes between high CREM expression and low CREM expression group in gastric cancer based on the TCGA GAC dataset. We used R package DESeq2 and filtered genes with base Mean >200, |log2FoldChange| > 1.5, *p*-value < 0.05. Finally, there were 723 up genes and 6,714 down genes.

### Functional Enrichment Analysis

In order to analyze the molecular mechanism of CREM in gastric cancer, we used these up genes in CREM high expression group to enrich in the KOBAS database (Xie et al., 2011) with the KEGG signaling pathway. We filtered these KEGG items with corrected p-value < 0.05. Besides, we also analyzed the TCGA GAC dataset with GSEA (gene set enrichment analysis) software (Subramanian et al., 2005) in KEGG pathways.

### Immune Cell Infiltration Analysis

We investigated every patient tumor-infiltrating immune cell abundance *via* the web-based tool Tumor Immune Estimation Resource (TIMER) version 2 (Li et al., 2020). We uploaded TCGA GAC TPM mRNA expression matrix in estimation web page and finally downloaded the estimated result. We analyzed the abundance of tumor-infiltrating immune cells with CIBERSORT (Chen et al., 2018). CIBERSORT was a mathematics method for characterizing cell composition of complex tissues including solid tumors *via* their gene expression profiles.

### Relationship Between CREM and T-Cell Exhaustion Analysis

In order to analyze the role of CREM in T cell’s function, we compared mRNA expression correlation between CREM and T cell’s exhausted marker genes in TCGA GAC datasets, such as HAVCR2, LAYN, TIGIT, PD1, and LAG3. Scatterplots were taken in GEPIA2 database with Spearman correlation coefficient. In addition, we defined T-cell exhausted score as CD8^+^ T-cell estimated infiltration abundance multiplied by the TPM expression value of T cell’s exhausted marker gene in every patient. Compared distribution of exhausted score between high CREM and low CREM groups. *p* < 0.05 was considered statistically significant (*p ≤ 0.05, **p ≤ 0.01, ***p ≤ 0.001, ****p ≤ 0.0001).

### Survival Analysis

Overall survival curves were taken by Kaplan–Meier plot of CREM high expression group and CREM low expression group with log rank test, and *p* < 0.05 was considered statistically significant (*p ≤ 0.05, **p ≤ 0.01, ***p ≤ 0.001, ****p ≤ 0.0001). Individual patients who did not have survival information were not considered in survival analysis. Besides, we explored overall survival of glioma and LUAD (lung adenocarcinoma) in the GEPIA2 web database. We chose GBM (glioblastoma) and LGG (brain lower grade glioma) as the glioma dataset; group was cut off by median.

## Results

### High CREM Expression in GAC Predicts Unfavorable Overall Survival

**FIGURE 1:**
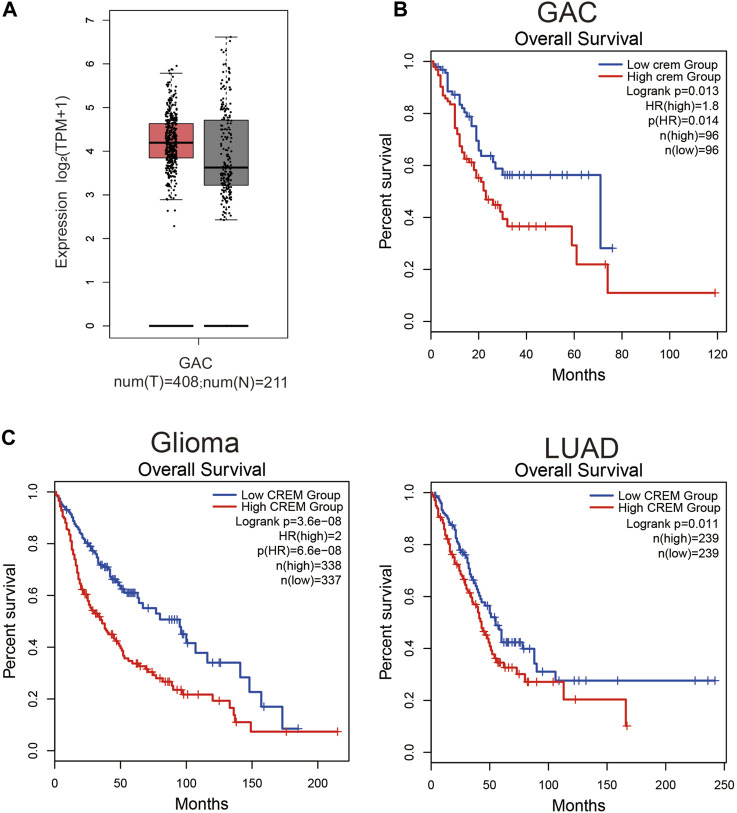
Expression level analysis of CREM in GAC tissues. Kaplan–Meier survival curves comparing the high and low expression of CREM in different types of cancer in GEPIA2. **(A)** CREM expression level between tumor samples and normal samples in GAC. **(B)** Kaplan–Meier survival curves comparing high and low CREM expression groups in the GAC dataset. **(C)** Kaplan–Meier survival curves comparing high and low CREM expression groups in the glioma, LUAD dataset.

To reveal the role of CREM in GAC, we investigated whether CREM expression was correlated with prognosis in GAC patients. By exploring CREM mRNA expression level and clinical characteristics of GAC in GEPIA2, the expression of CREM in GAC was higher than that in normal tissues (Figure 1A). CREM is highly expressed in tumor tissues and may play an important role in GAC. To better understand the potential function of CREM in GAC, we analyzed the relationship between the CREM expression and clinical characteristics of GC patients in the TCGA database. GAC patients with higher CREM expression had shorter overall survival (*p* = 0.013) (Figure 1B). These results indicated that CREM was frequently expressed in GAC and could serve as a prognostic biomarker in GAC. Notably, CREM expression significantly impacts prognosis in gliomas and LUAD. LUAD patients and glioma patients with higher CREM expression had shorter overall survival (Figure 1C). These results indicated that CREM expression is an independent risk factor and leads to a poor prognosis in GAC patients.

### CREM Was Involved in Many Cancer-Associated Signaling Pathways

**FIGURE 2:**
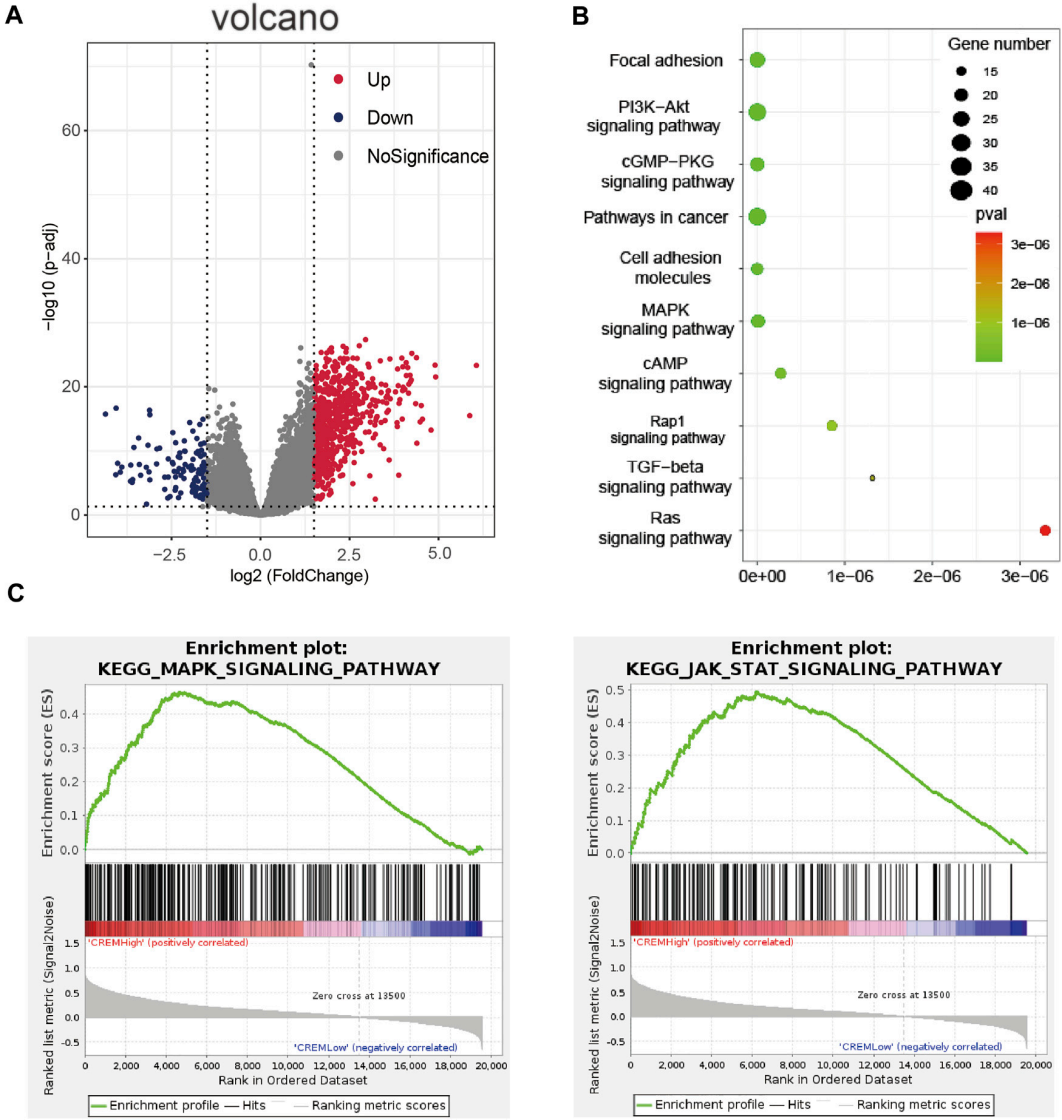
Enrichment analyses of CREM in TCGA GAC (stomach adenocarcinoma). **(A)** Volcano map of different expression genes between high expression CREM group and low expression CREM group. Red represents upregulation of gene expression, purple represents downregulation of gene expression, and gray represents no difference in gene expression. **(B)** Enrichment analysis of CREM different expression up genes in KOBAS database with KEGG pathways. Plot sizes show gene counts enriched in the enrichment of pathway. Color indicates the p-value from low (red) to high level (cyan). **(C)** Enrichment plot from gene set enrichment analysis including enrichment score. The significantly enriched signaling pathways were MAPK and JAK-STAT signaling pathway.

In order to further verify the role of CREM in GAC, by analyzing TCGA database, we cluster the genes with high and low expression of CREM (Figure 2A, Supplementary Table S1). These genes with high expression of CREM were clustered into the KEGG pathway by the KOBAS database (Figure 2B). We found that CREM is associated with a variety of cancer-associated pathways, such as focal adhesion, PI3K-Akt signaling pathway, cGMP-PKG signaling, cell adhesion molecules, MAPK signaling pathway, Rap1 signaling pathway, TGF-beta signaling pathway, and Ras signaling pathway. Notably, we also analyzed KEGG pathway in GSEA software. In the CREM overexpression group, MAPK signaling pathway and JAK-STAT signaling pathway were enriched (Figure 2C). MAPK signaling pathway plays a crucial role in the survival and development of tumor cells. The JAK-STAT signaling pathway also plays the external role of tumor and supports tumor survival by regulation of paracrine cytokine signaling. The identity of these tumor-related pathways in CREM high expression group proved that the high expression of CREM in tumor and TME did change the characteristics of tumor, and the high expression of CREM in tumor tissues led to the activation of cancer-associated signaling pathways and poor prognosis.

### CREM is Positively Correlated With Exhausted T-Cell Marker Genes in GAC

**FIGURE 3:**
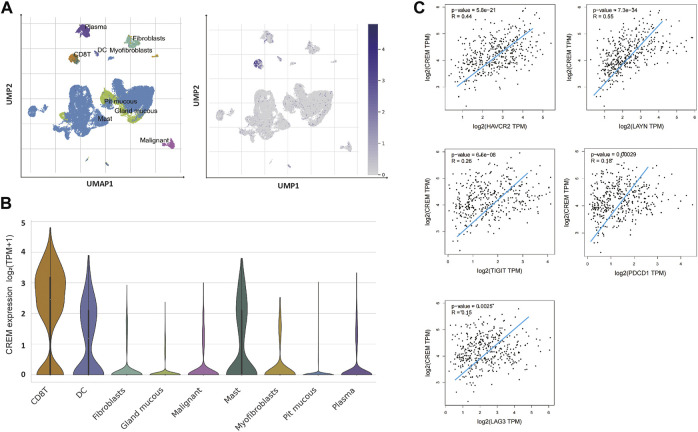
The CREM expression analysis of tumor-infiltrating lymphocytes in GAC. **(A)** UMAP of GAC tumor tissue single-cell dataset, UMAP of GAC single cell dataset showed that high expression of CREM in CD8+ T cells. The depth of blue represents the amount of gene expression. **(B)** Violin plots showing the expression of CREM in various cell types. **(C)** Relationship between CREM expression level and T-cell exhausted marker genes expression level in the TCGA database (gene expression value is log2 TPM).

**FIGURE 4:**
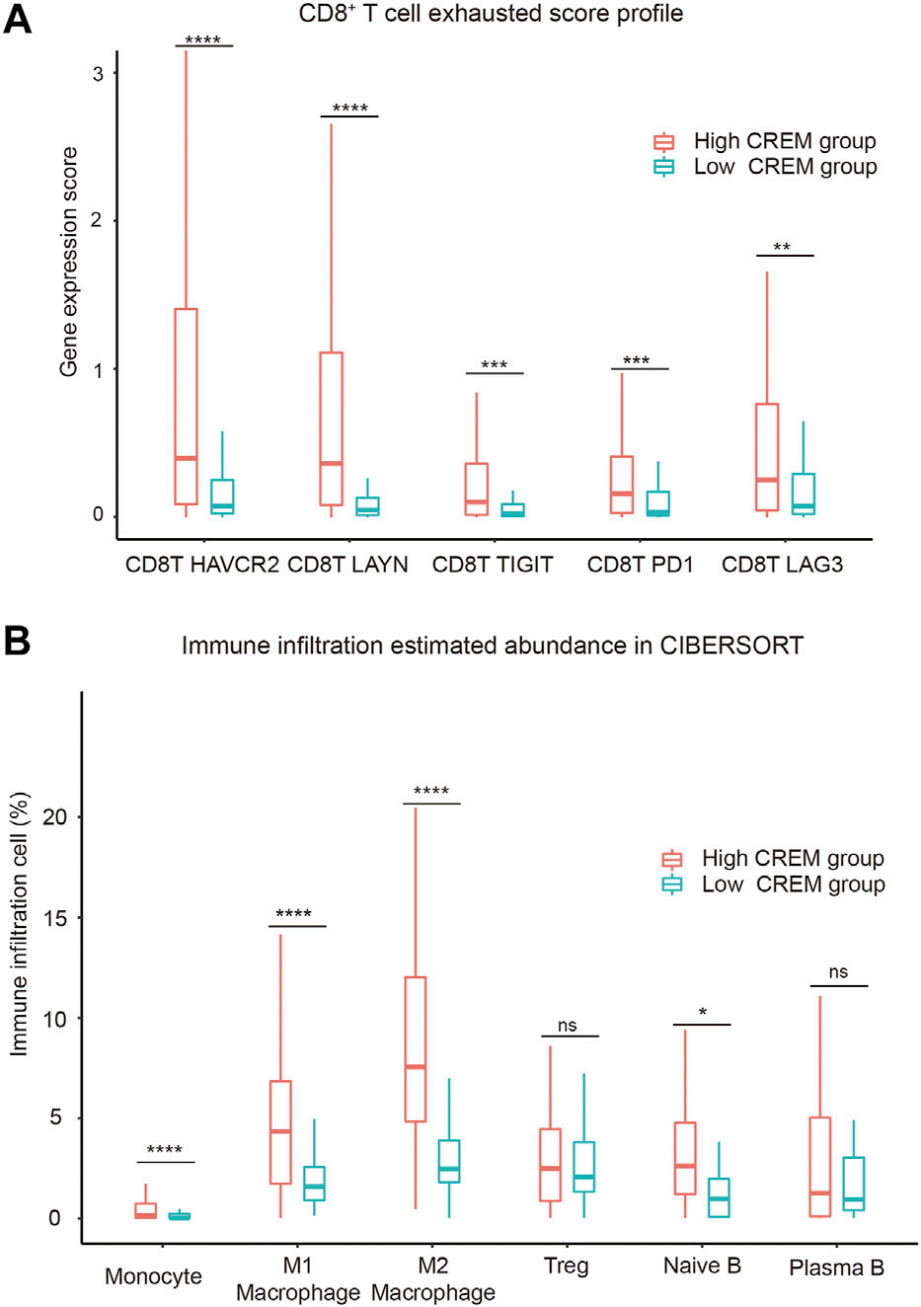
Correlation between CREM expression and immune-suppressive microenvironment. **(A)** CD8^+^ T-cell exhausted score profile between the high CREM group and low CREM group. **(B)** The immune infiltration estimated abundance in CIBERSORT; the estimated abundance is absolute type in CIBERSORT method.

In order to further explore the potential reasons for the positive correlation between CREM and tumorigenesis and development, we further investigated the correlation between CREM expression with tumor immune microenvironment. T-cell exhaustion is a state of T-cell dysfunction that arises during cancer and chronic inflammation in immune microenvironment. It is defined by poor function and sustained expression of inhibitory receptors. T-cell exhaustion prevents optimal control of infection and tumor progression. Previous studies have confirmed that CREM can affect T-cell function. In our study, we questioned whether the effect of CREM on poor prognosis in cancer was associated with immune-suppressive microenvironment. To investigate the cause of poor prognosis in GAC with high expression of CREM, we further explored CREM mRNA expression profile in the TISCH single-cell database in GC. We found that CREM can be expressed in a variety of cells, such as CD8^+^ T cells, DC cells, mast cells, myofibroblasts, malignant cells, and fibroblasts. Notably, CREM is highly expressed in CD8^+^ T cells in TME (Figures 3A,B). Furthermore, we speculate that CREM may affect T-cell function in TME. To evaluate whether the high expression of CREM affects the function of T cells in GAC, we compared the gene expression levels of CREM and exhausted T-cell marker genes (TIM3, TIGIT, LAG3, PD1, and LAYN). Our results indicated that CREM was significantly correlated with exhausted T-cell marker genes (Figure 3D). In addition, in the high CREM group, the number of estimated exhausted T cells increased significantly (Figure 4A). Overall, these results indicated that CREM is associated with CD8^+^ T-cell exhaustion in GAC. Notably, we analyzed the role of CREM in lung cancer and glioma. CD4^+^ CTLA4 and CD8^+^ LAYN are considered to be part of the T-cell exhaustion population. Single-cell sequencing data showed that CREM is highly expressed in the CD4^+^ CTLA4 and CD8^+^ LAYN population in non-small cell lung cancer (Supplementary Figure S1C), and CREM is highly expressed in the CD8^+^ T-cell exhaustion population in glioma (Supplementary Figure S1B). These findings suggest that CREM plays a specific role in T-cell exhaustion in GAC and may affect the exhaustion of T cell in lung cancer and glioma.

### The Role of CREM Expression on the Abundance of Immune Cell Infiltration

Tumor-infiltrating lymphocytes are an independent predictor of survival in cancer (Stanton and Disis, 2016; Badalamenti et al., 2019). To assess if high CREM expression was correlated with immune infiltration levels in GC, CIBERSORT was used to analyze the infiltration abundance of several immune cells between the high CREM group and low CREM group. Our results showed that the infiltration rates of M1 macrophages and M2 macrophages were significantly higher in the CREM high expression group. Treg cells (regulatory T cells) and plasma B cells had no significant changes. Interestingly, there was more infiltration of M1 and M2 macrophages in the CREM overexpression group, while the infiltration of M2 macrophages was more obvious than that of M1 macrophages or other immune cells (Figure 4B). These results indicated that CREM was positively correlated with the M2 polarization of macrophage in GAC. In general, these findings indicated that CREM-mediated immunosuppression may attribute to M2 polarization of TAMs.

## Discussion

Most malignant tumors are immunologically silent. In order to grow in an immune-competent host, acquired tumor cells undergo changes that result in immune-resistant phenotypes (Jiang et al., 2020). The high expression of CREM in tumor and TME may be a way for tumors to fight immune cells. CREM is part of the cAMP responsive element modulator family responsible for regulation of gene expression. Our study depicts the relationship between CREM and cancer-associated signaling pathway, and the immune features associated with CREM in the TME of GAC.

Higher CREM expression predicted poor outcome in patients with GAC, glioma, and LUAD. We found different expression genes between high CREM expression and low in the GAC TCGA dataset. The GSEA analysis revealed that the CREM expression level was strongly positively correlated with the KEGG MAPK signaling pathway and the JAK-STAT pathway. These results indicated that the high expression of CREM in tumor tissue led to the activation of the cancer-associated pathway in tumor cells and tumor progression.

Of note, our results revealed that CREM expression is associated with the exhausted T cells and infiltration of immune-suppressive macrophages in GAC, indicating a critical role of CREM in modulating the immune-suppressive microenvironment. The previous study provided evidence that CREM prevents production of IL-2 during chronic viral infection, thereby contributing to T-cell exhaustion (Maine et al., 2016). T-cell exhaustion refers to the loss of effector function of T cells, such as decreased production of IL-2 and IFN-γ, and expression of PD-1, Tim-3, CTLA-4, LAG-3, and other inhibitory receptors. We compared the expression levels of CREM and T-cell inhibitory receptors in GAC and found that CREM expression was positively correlated with the expression of T-cell suppressor molecules. On the other hand, M1 macrophages are associated with anti-tumor properties that efficiently eliminate cancer cells through phagocytosis and cytotoxicity while M2 macrophages promote tissue repair and tumor growth. The increased presence of M1 macrophages denotes lower tumor malignancy, while a higher M2 presence causes increased tumor growth and decreased survival (Ruffell et al., 2012; Xia et al., 2020). We analyzed and compared the proportion of tumor-infiltrating lymphocytes in GAC between the high CREM group and the low CREM group. Significantly, the results showed that CREM high expression was significantly correlated with M2 polarization of microphage. These results support the notion that the high expression of CREM in tumor-infiltrating T cells leads to the development of immune-suppressive microenvironment, activation of tumor-related pathways, and proliferation and development of tumor cells. These results reveal the potential regulating role of CREM in polarization of tumor-associated macrophages. The above indicated that CREM could serve as an important prognostic risk factor for GAC, and this study may provide a new biomarker for immunotherapy of GAC.

Experimental studies are needed to validate the findings of this study. The experimental validation of the predicted results by different methods should be further confirmed. Lastly, it is not clear whether the immune-suppressive microenvironment would induce CREM expression or *vice versa*, and further research is needed. This study provides preliminary results that CREM is correlated with T-cell exhaustion and M2 polarization of macrophages in GAC.

## Data Availability

Publicly available datasets were analyzed in this study. This data can be found here: https://gdc-hub.s3.us-east-1.amazonaws.com/download/TCGA-STAD.htseq_counts.tsv.gz http://lung.cancer-pku.cn.

## Supplementary Material

Supplementary Figure S1 The CREM expression analysis of tumor infiltrating lymphocytes in glioma and non-small cell lung cancer in single cell dataset. **(A)** Cluster profile of glioma single cell dataset in glioma GSE131928. **(B)** CREM expression levels of different cell types in glioma single cell dataset. **(C)** CREM expression levels of different T cell populations in the single cell data set of non-small cell lung cancer. **(D)** Cluster profile of T cells in non-small cell lung cancer dataset in web database (http://lung.cancer-pku.cn).

Supplementary Table S1 Table of differential expression genes.
