# Supplementary figures and images for: CREM Is Correlated With Immune-Suppressive Microenvironment and Predicts Poor Prognosis in Gastric Adenocarcinoma
Source: Front Cell Dev Biol. 2021 Dec 6;9:697748. doi: 10.3389/fcell.2021.697748 (PMC8685542; doi:10.3389/fcell.2021.697748)

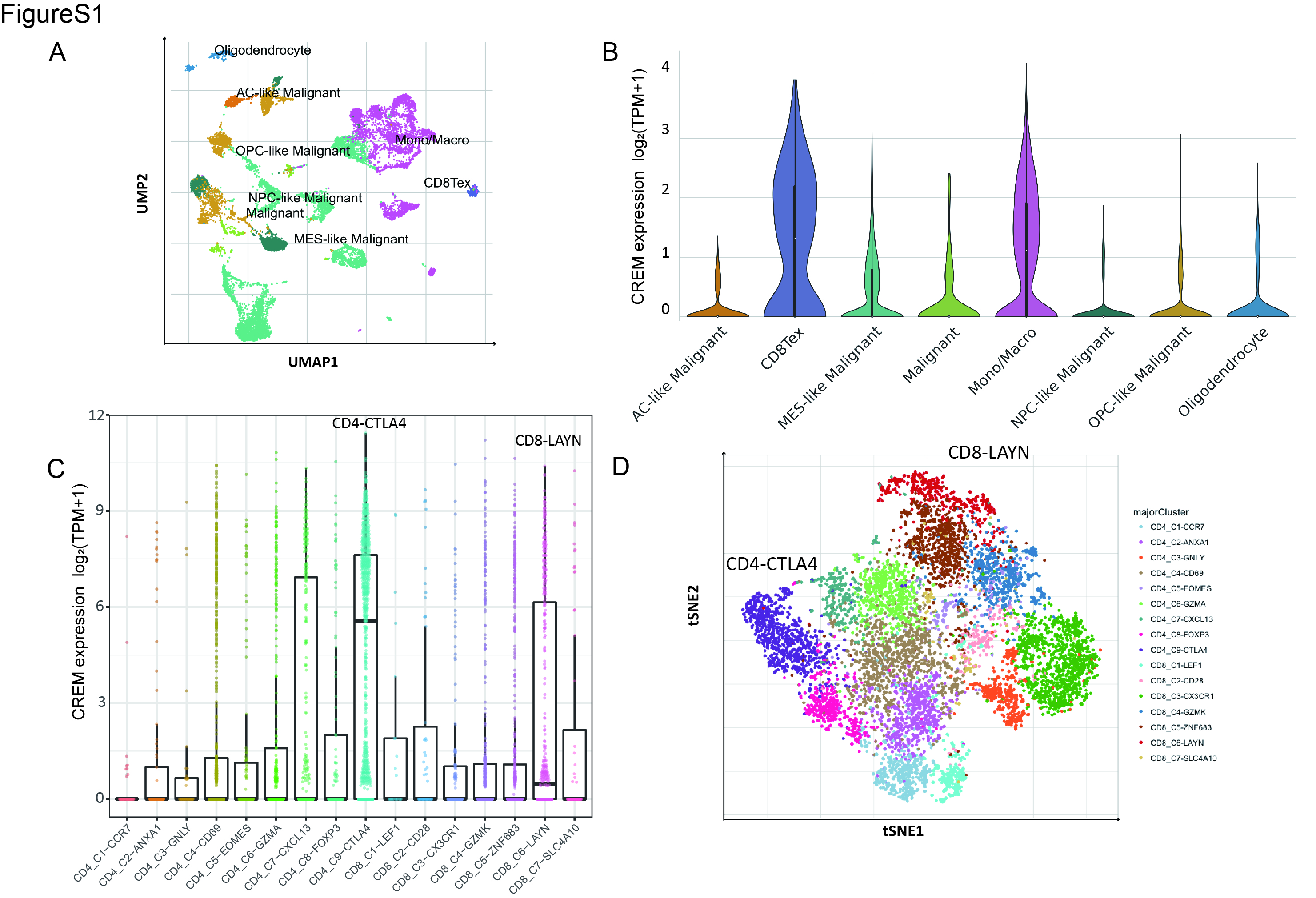

Supplement: Supplementary file 2 [file Image1.jpeg]
